# Cardiac drug-drug interaction between HCV-NS5B pronucleotide inhibitors and amiodarone is determined by their specific diastereochemistry

**DOI:** 10.1038/srep44820

**Published:** 2017-03-22

**Authors:** Armando Lagrutta, Christopher P. Regan, Haoyu Zeng, John P. Imredy, Kenneth Koeplinger, Pierre Morissette, Liping Liu, Gordon Wollenberg, Christopher Brynczka, José Lebrón, Joseph DeGeorge, Frederick Sannajust

**Affiliations:** 1 Dept. Safety and Exploratory Pharmacology, Safety Assessment and Laboratory Animal Resources, MRL, Merck & Co., West Point, PA, USA; 2 Dept. Preclinical ADME, Pharmacokinetics, Pharmacodynamics and Drug Metabolism, MRL, Merck & Co., West Point, PA, USA; 3 Dept. Investigative Laboratory Sciences, Safety Assessment and Laboratory Animal Resources, MRL, Merck & Co., West Point, PA, USA; 4 Dept. Pathology, Safety Assessment and Laboratory Animal Resources, MRL, Merck & Co., West Point, PA, USA; 5 Dept. Program Development, Safety Assessment and Laboratory Animal Resources, MRL, Merck & Co., West Point, PA, USA; 6 Safety Assessment and Laboratory Animal Resources, MRL, Merck & Co., West Point, PA, USA

## Abstract

Severe bradycardia/bradyarrhythmia following coadministration of the HCV-NS5B prodrug sofosbuvir with amiodarone was recently reported. Our previous preclinical *in vivo* experiments demonstrated that only certain HCV-NS5B prodrugs elicit bradycardia when combined with amiodarone. In this study, we evaluate the impact of HCV-NS5B prodrug phosphoramidate diastereochemistry (D-/L-alanine, R-/S-phosphoryl) *in vitro* and *in vivo*. Co-applied with amiodarone, L-ala,*S*_P_ prodrugs increased beating rate and decreased beat amplitude in human induced pluripotent stem cell-derived cardiomyocytes (hiPSC-CMs), but D-ala,*R*_P_ produgs, including MK-3682, did not. Stereochemical selectivity on emerging bradycardia was confirmed *in vivo*. Diastereomer pairs entered cells equally well, and there was no difference in intracellular accumulation of L-ala,*S*_P_ metabolites ± amiodarone, but no D-ala,*R*_P_ metabolites were detected. Cathepsin A (CatA) inhibitors attenuated L-ala,*S*_P_ prodrug metabolite formation, yet exacerbated L-ala,*S*_P_ + amiodarone effects, implicating the prodrugs in these effects. Experiments indicate that pharmacological effects and metabolic conversion to UTP analog are L-ala,*S*_P_ prodrug-dependent in cardiomyocytes.

Cardiac safety concerns about intracellular accumulation of nucleos(t)ide inhibitors arose in the context of failed clinical trials, with the hepatitis C virus nonstructural protein 5B (HCV-NS5B) inhibitor BMS-986094^1^,^2^. Since then, investigations have highlighted putative mechanisms of nucleotide-based toxicities, and new approaches for de-risking have been developed using cellular and biochemical methodologies^3^. Recent reports of severe bradycardic/proarrhythmic effects following coadministration of the uridine nucleotide analog prodrug sofosbuvir with the class-III antiarrhythmic amiodarone, and perhaps other co-administered drugs, including several direct-acting antiviral agents (DAAs), have triggered new cardiac toxicity safety concerns and more detailed preclinical/clinical investigations^4^. While early reports and editorials suggested these clinical adverse events are mediated by common pharmacokinetic drug-drug interactions (DDI), preclinical experiments in human induced pluripotent stem cell-derived cardiomyocytes (hiPSC-CMs) implicated a novel pharmacodynamic interaction between sofosbuvir and amiodarone, related to potential disruptions in intracellular Ca^2+^ handling mechanisms^5^,^6^,^7^. Moreover, these observations were extended to Merck Nucleotide Inhibitor-1 (MNI-1), a pronucleotide inhibitor tool compound structurally related to sofosbuvir^5^. Further, *in vivo* experiments recapitulating the sofosbuvir:amiodarone clinical cardiac effects have demonstrated that not all HCV-NS5B nucleotide prodrugs share the same DDI liability. Specifically, bradycardia and bradyarrhythmia were not observed in rhesus monkeys when intravenous infusion of MK-3682 was completed after AMIO pretreatment^7^. In this study, we examine the putative role played by the stereochemistry of the amino acyl and phosphoryl group of HCV-NS5B phosphoramidate prodrugs in the adverse cardiac DDI with amiodarone. To this end, we have systematically compared three uridine analog HCV-NS5B inhibitor prodrugs that display D-ala,*R*_P_ stereochemistry (Merck nucleotide inhibitors MNI-2, MNI-4 and MK-3682) with their L-ala,*S*_P_ counterparts (sofosbuvir, MNI-1, and MNI-3, respectively).

A preferred stereochemistry imparting greater safety to phosphoramidate prodrugs could be related to the stereoselectivity of intracellular converting enzymes^8^,^9^. Murakami and colleagues showed stereoselectivity of Cathepsin-A (CatA), favoring the L-ala,*S*_P_ diastereoisomer, and of CES1, favoring the L-ala,*R*_P_ diastereoisomer^10^. In addition, the authors demonstrated the L-ala,*S*_P_ stereochemical configuration provided much greater catalytic efficiency in the clone A replicon assay compared to the L-ala,*R*_P_ diastereoisomer or compared to the mixture of the two phosphate diastereoisomers, but this was related to selective expression of CatA and little or no expression of CES1^10^. In contrast, little difference was observed in nucleotide triphosphate (NTP) activation in primary human hepatocytes when comparing either phosphate diastereoisomer or the mixture^10^. Together, these findings indicated redundancy between CatA and CES1 in the metabolism of phosphoramidate prodrugs, but stereochemical specificty in metabolism of the drug by CatA and CES1.

An additional consideration is the tissue-specific expression of intracellular converting enzymes, best characterized in liver or small intestine, involved in first-pass metabolism^8^,^11^,^12^. Current understanding suggests the metabolism of sofosbuvir, and other phosphoramidate L-ala,*S*_P_ HCV-NS5B nucleotide prodrugs by CatA and CES1 inside hepatocytes results in the formation of a cleavage intermediate metabolite which can be further metabolized by Histidine triad Nucleotide-Binding Protein 1 (HINT-1) to the nucleotide monophosphate. The nucleotide monophosphate is then phosphorylated by intracellular kinases to form the pharmacologically active NTP^13^,^14^. Similarly, active NTP metabolite formation from phosphoramidate D-ala,*R*_P_ prodrugs of uridine monophosphate analogs, such as MK-3682, is via the formation of a cleavage intermediate metabolite that is further metabolized by HINT-1 to the uridine monophosphate analog and subsequently phosphorylated by intracellular kinases to form the pharmacologically active NTP. Cardiac-specific differences in NTP analog accumulation, based on stereochemistry and tissue-restricted expression of enzymes required for production of metabolites, may therefore play a role in the adverse DDI. Alternatively, one could envision an analogous stereochemical interaction between the prodrug and a target completely unrelated to carboxylesterases or NTP conversion, yet to be characterized, that might underlie the adverse cardiac DDI.

## Results

### Stereochemical specificy of interaction between hepatitis C Virus HCV-NS5B inhibitors and amiodarone in hiPSC-CM syncytia model

**Figure 1:**
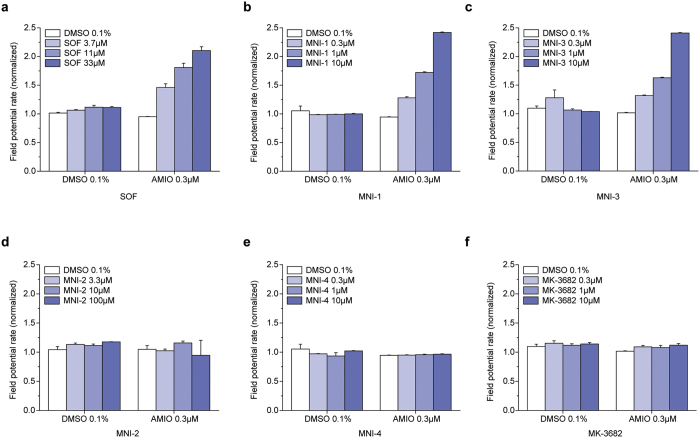
Concentration-dependent, steady-state effects by L-ala,*S*_P_ prodrugs (SOF, MNI-1, MNI-3) and lack of effects by D-ala,*R*_P_ prodrugs (MNI-2, MNI-4, MK-3682) co-administered with 0.3 μM amiodarone on FP rate measured in spontaneously beating hiPSC-CM syncytia. (**a–f**) Colored bar graphs in each panel show steady state effects produced by increasing concentrations of each prodrug ± amiodarone: (**a**) Sofosbuvir (SOF). (**b**) MNI-1. (**c**) MNI-3. (**d**) MNI-2. (**e**) MNI-4. (**f**) MK-3682. (**a–f**) Clear bar graphs illustrate measurement with DMSO vehicle or amiodarone alone. Data are normalized to parameters measured at time = 0 (mean ± SEM, n = 6).

**Figure 2:**
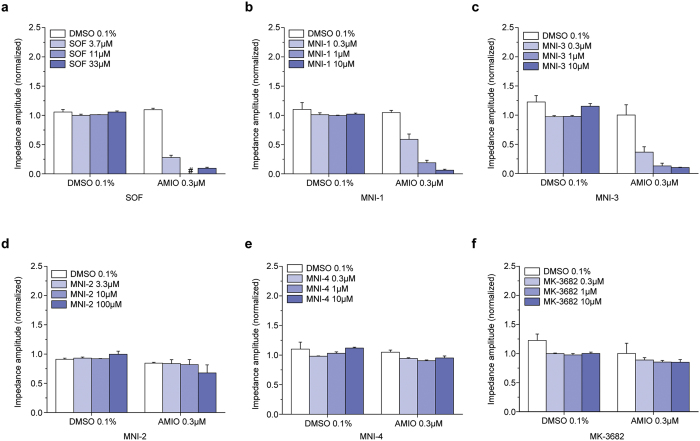
Concentration-dependent, steady-state effects by L-ala,*S*_P_ prodrugs (SOF, MNI-1, MNI-3) and lack of effects by D-ala,*R*_P_ prodrugs (MNI-2, MNI-4, MK-3682) co-administered with 0.3 μM amiodarone on IMP amplitude measured in spontaneously beating hiPSC-CM syncytia. (**a–f**) Colored bar graphs in each panel show steady state effects produced by increasing concentrations of each prodrug ± amiodarone: (**a**) Sofosbuvir (SOF). (**b**) MNI-1. (**c**) MNI-3. (**d**) MNI-2. (**e**) MNI-4. (**f**) MK-3682. (**a**–**f**) Clear bar graphs illustrate measurement with DMSO vehicle or amiodarone alone. Data are normalized to parameters measured at time = 0. (mean ± SEM, n = 3). The effect by 11 μM sofosbuvir co-applied with 0.3 μM amiodarone was below the limit of detection (#).

We have previously reported the effect of sofosbuvir or a closely related analog, designated as MNI-1, when co-applied with amiodarone, on the spontaneous beating rate and amplitude of hiPSC-CM syncytia^5^. Similarly, we have reported how these drugs, when coadministered with amiodarone, produce emergent bradycardia in anesthetized guinea pigs and bradycardia/bradyarrhythmia in conscious, chair-restrained rhesus monkeys^7^. We used these models to help understand the basis of a clinically observed cardiac DDI associated with severe bradycardia. In this study, we compare systematically three pairs of HCV-NS5B prodrugs, with L-ala,*S*_P_ or D-ala,*R*_P_ diastereochemistry: sofosbuvir vs. MNI-2, MNI-1 vs. MNI-4, and MNI-3 vs. MK-3682. Table 1 summarizes the structures of each studied HCV-NS5B prodrug, as well as structures of respective cleavage intermediate metabolites and NTP metabolites that were measured intracellularly. We have observed concentration-dependent effects, at concentrations that approximate the clinical or projected clinical C_max_ of amiodarone and each HCV-NS5B prodrug, on spontaneous field potential (FP) rate (Fig. 1a–f) and on impedance (IMP) amplitude (Fig. 2a–f) of hiPSC-CM syncytia, when phosphoramidate L-ala,*S*_P_ 2′-methyl ribose substituted nucleotide prodrugs were co-applied with amiodarone, and lack of these effects by the corresponding phosphoramidate D-ala,*R*_P_ 2′-methyl ribose substituted nucleotide prodrugs when co-applied with amiodarone. The measurements shown are steady-state effects at 4 h. As previously reported, the observations in this hiPSC-CM model are paradoxical increases in FP rate, instead of decreases, accompanied by decreases in IMP amplitude^5^,^6^. Furthermore, we studied the effects on FP rate and IMP amplitude of “mixed” diastereoisomeric phosphoramidate prodrugs, sharing the nucleoside analog structure of sofosbuvir and MNI-2, but either L-ala,*S*_P_ (MNI-5) or D-ala,*R*_P_ (MNI-6) in their stereochemical configuration (structures shown on Supplementary Table 1). Neither one of these prodrugs produced changes in spontaneous FP rate or IMP amplitude in hiPSC-CM syncytia, alone or when combined with amiodarone (Supplementary Fig. 1a,b).

### Determination of prodrug, cleavage intermediate metabolite, and NTP metabolite concentration in hiPSC-CMs

Table 2 summarizes the concentrations of prodrug, cleavage intermediate metabolite and NTP metabolite extracted from hiPSC-CMs incubated in multi-well plates for 30 min and 4 h with various phosphoramidate L-ala,*S*_P_ and D-ala,*R*_P_ 2′-ribose substituted HCV-NS5B nucleotide prodrugs, alone (10 μM) or in combination with amiodarone (0.3 μM). Each experimental condition represents mean ± SEM (n = 3). For sofosbuvir and MNI-1, L-ala,*S*_P_ prodrugs, the concentration of cleavage intermediate metabolite accumulated to 8–10 fold of prodrug concentration after 4 h. For MNI-3, the other L-ala,*S*_P_ prodrug examined, the concentration of cleavage intermediate metabolite accumulated to 50 fold of prodrug concentration after 4 h. Concentrations of NTP metabolite were undetectable at 30 min, increasing by 4 h, as shown on Table 2. In contrast, there were no detectable levels of cleavage intermediate metabolite or NTP metabolite following application of any of the D-ala,*R*_P_ prodrugs. Coadministration of amiodarone showed no profound impact on the concentrations of L-ala,*S*_P_ prodrug, cleavage intermediate metabolite or NTP metabolite, or in the concentrations of D-ala,*R*_P_ prodrug measured, conmensurate with the observed FP rate or IMP amplitude effects (Figs 1a–f and 2a–f).

**Figure 3:**
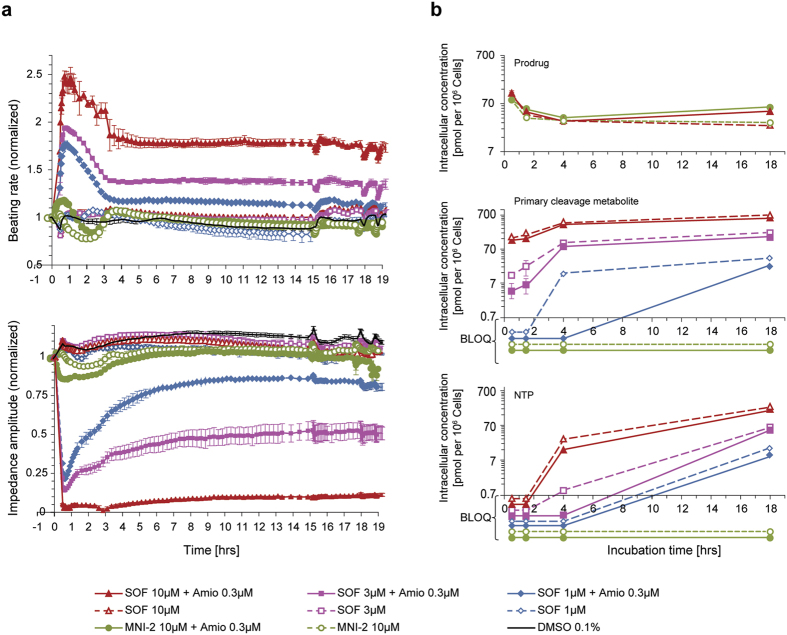
Pharmacokinetic-pharmacodynamic relationships for sofosbuvir (1, 3, 10 μM) or MNI-2 prodrug (10 μM) ± amiodarone (0.3 μM) in hiPSC-CM syncytia. (**a**) Effects on beating rate and amplitude, monitored for the various conditions on the fluctuating IMP signal in the RTCA Cardio platform for up to 18 h. Data are normalized to parameters measured at time = 0 (mean ± SEM, n = 3). (**b**) Concentrations of prodrugs (top), cleavage intermediate metabolite (middle) and NTP (bottom). Cardiomyocytes were harvested for prodrug or metabolite extraction at 0.5 h, 1.5 h, 4 h, and 18 h post-application of prodrugs, alone and/or combined with amiodarone (mean ± SEM, n = 3). Transient overshoots over steady state responses (**a**) and initial prodrug accumulation over steady state levels (**b**) could be related to the application of prodrug as a 37X bolus in this particular study. Measurements below the level of quantitation (BLOQ, panel b) are shown at the appropriate time points, simply to illustrate that they were attempted.

We directly compared the time courses of the pharmacodynamic effects (Fig. 3a) and pharmacokinetic effects (Fig. 3b) of sofosbuvir (1.0, 3.0, and 10.0 μM) and its D-ala,*R*_P_ counterpart, MNI-2 (10 μM), alone or in combination with amiodarone (0.3 μM) over an 18-h period. Pharmacokinetic and pharmacodynamic measurements (beating rate and amplitude monitored by the fluctuating IMP signal in RTCA Cardio platform) were obtained from the same hiPSC-CM plates. Spontaneously beating hiPSC-CM syncytia were monitored for 0.5 h, 1.5 h, 4 h, and 18 h prior to harvest for analysis of intracellular accumulation of the prodrug, the cleavage intermediate metabolite, and NTP. Figure 3a plots the time course of effects on IMP-measured beating rate and IMP amplitude for the various conditions tested. The D-ala,*R*_P_ prodrug MNI-2 combined with amiodarone failed to evoke changes over each drug alone. In the presence of 0.3 μM amiodarone, the L-ala,*S*_P_ prodrug sofosbuvir effected robust, concentration-dependent changes in both beating rate (+146% ± 11% at 10 μM) and amplitude (−97% ± 2% at 10 μM). Figure 3b (top) shows comparable intracellular accumulation of the two prodrugs applied at the same concentration (10 μM). Figure 3b also shows the accumulated intracellular concentrations of the cleavage intermediate metabolite (middle) and final NTP (bottom). As mentioned above, no measurable concentrations of cleavage intermediate metabolite or NTP were observed following application of MNI-2, suggesting a lack of metabolism for this D-ala,*R*_P_ prodrug in hiPSC-CMs. When intracellular concentrations of sofosbuvir and metabolites were compared to the time course of the sofosbuvir + amiodarone dependent beating rate and amplitude measured by fluctuating IMP in the RTCA Cardio platform, the sofosbuvir prodrug concentration displayed the most closely matching pharmacokinetic time course. The steadily rising concentrations of the cleavage intermediate metabolite, and the late onset of measurable accumulation of NTP over the 18-h time course, argue for a putative secondary role for these metabolites in the observed pharmacodynamic effects.

In addition, when the “mixed” stereoisomers of sofosbuvir, MNI-5 and MNI-6, described in the previous section, were studied for their ability to accumulate cleavage intermediate metabolite or NTP metabolite, we observed that prodrug levels were accumulated to similar levels than sofosbuvir or MNI-2 (Supplementary Table 1). Surprisingly, we observed that concentration of cleavage metabolite intermediate for one of these prodrugs, MNI-5, L-ala,*R*_P_ in its stereochemistry, reached measurable levels after 4-h incubation, albeit ~80% reduced compared to those accumulated by incubation of sofosbuvir, its corresponding L-ala,*S*_P_ prodrug, yet distinct from levels below the limit of quantitation detected for MNI-2, its corresponding D-ala,*R*_P_ prodrug (Table 2 and Supplementary Table 1).

### Coadministration of MNI-2 and amiodarone did not cause bradycardia *in vivo*

**Figure 4:**
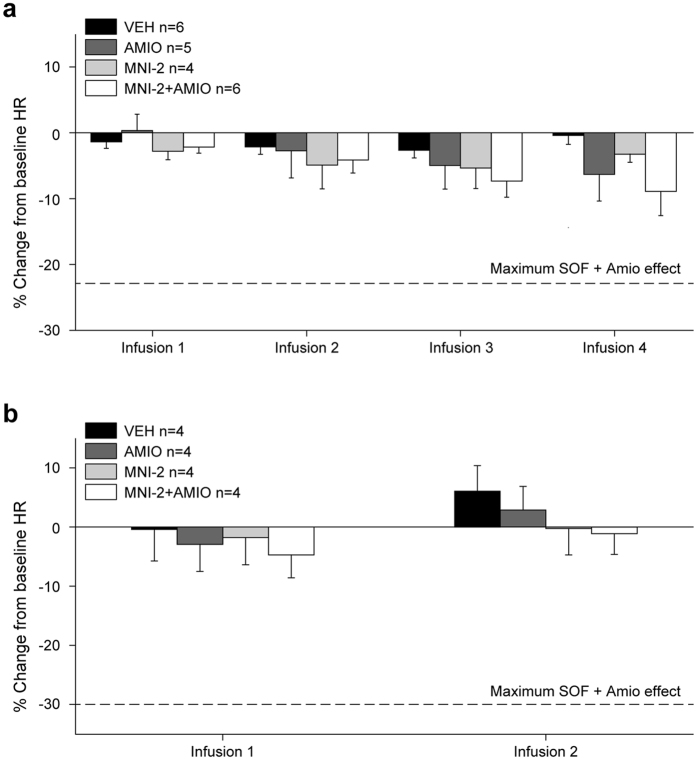
Coadministration of MNI-2 and amiodarone did not cause bradycardia *in vivo*. (**a**) Effects of successive 30-min IV infusions of MNI-2 alone (4, 2, 2, then 2 mg/kg/30 min) or in combination with amiodarone (1, 0.5, 0.5, thn 0.5 mg/kg/30 min), on HR (expressed as a % change [%CH] from baseline) in anesthetized, male guinea pigs. (**b**) Effects of IV infusion of MNI-2 (4, then 2 mg/kg/30 min) alone or after prior administration of IV amiodarone (AMIO, 3 mg/kg/30 min), expressed as a % change [%CH] from baseline) in conscious, male rhesus monkeys. Dotted line represents the maximum % change from baseline in HR as previously reported^7^.

The heart rate (HR) effects of MNI-2 alone or in combination with amiodarone were evaluated in ketamine/xylazine-anesthetized, male Dunkin-Hartley guinea pigs. Administration of cumulative IV infusion dose of 2.5 mg/kg amiodarone alone or 10 mg/kg MNI-2 alone decreased HR by −7% and −3% (% change from baseline), respectively (Fig. 4a). Coadministration of these IV doses of MNI-2 and amiodarone did not result in a greater decrease in HR than predicted by either agent alone (−9% vs.−7% and −3% for each agent alone). Plasma levels of MNI-2, MNI-2 cleavage intermediate metabolite, and amiodarone were not different among treatment groups (Supplementary Table 2).

There was no effect of IV infusion of MNI-2, alone or after prior administration of amiodarone, on HR in conscious male, restrained rhesus monkeys (Fig. 4b). Plasma concentration of MNI-2 and amiodarone at the end of the MNI-2+amiodarone experiment were 3048 ± 413 ng/mL and 402 ± 23 ng/mL, respectively. In addition, IV infusion of MNI-2 alone or after pretreatment with amiodarone did not result in MBP or PR, QRS, QT ECG interval changes (data not shown).

Furthermore, cardiac tissues from anesthetized guinea pigs were collected immediately following the end of the MNI-2 and MNI-2+amiodarone IV infusions to evaluate tissue concentrations of MNI-2 parent prodrug, MNI-2 cleavage intermediate metabolite, and MNI-2-NTP metabolite (Supplementary Fig. 1). We previously showed that cardiac sofosbuvir concentrations were not higher in anesthetized guinea pigs following cummulative IV administration of sofosbuvir or sofosbuvir + amiodarone (~6 nmol/g tissue) compared to sofosbuvir alone (~8 nmol/g tissue)^7^. In agreement with the *in vitro* data on hiPSC-CM syncytia, D-ala,*R*_P_ MNI-2 did not produce detectable levels of cleavage intermediate metabolite or NTP metabolite, while L-ala,*S*_P_ sofosbuvir did (Supplementary Fig. 1). Finally, sofosbuvir cleavage intermediate and NTP metabolites were not higher in cardiac tissues from anesthetized guinea pigs administered sofosbuvir + amiodarone vs. sofosbuvir alone (Supplementary Fig 1).

### Effect of CatA inhibitors in hiPSC-CM syncytia

**Figure 5:**
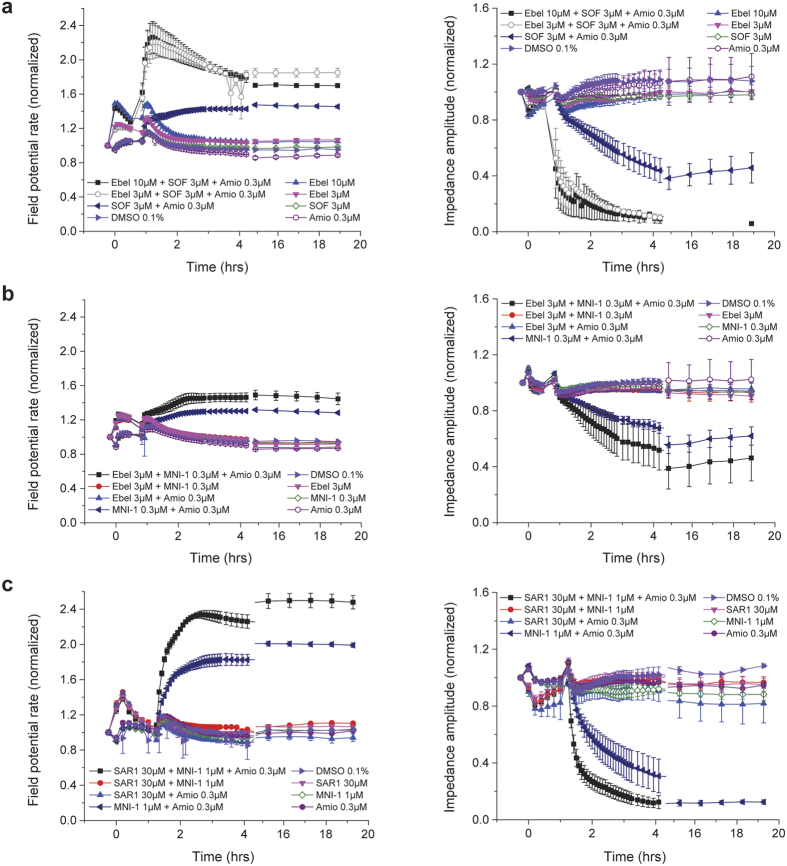
Effect of CatA inhibitor pre-incubation and continued incubation on HCV prodrug DDI in hiPSC-CM syncytia. (**a**) Effects of Ebel on the DDI between sofosbuvir+amiodarone. (**b**) Effects of Ebel on the DDI between MNI-1 + amiodarone. (**c**) Effect of CatA inhibitor SAR164653 (SAR1) on the DDI between MNI-1 + amiodarone. (**a–c**) FP rate (left) and IMP amplitude (right) were monitored, measured and normalized to the responses at time = 0. Appropriate controls in each panel show the baseline effects of DMSO vehicle alone, amiodarone alone, and HCV NI prodrug alone.

Figure 5 shows the effects of CatA inhibitor ebelactone B (Ebel) and a novel, more selective CatA inhibitor, designated as compound 2a, SAR1, or SAR164653^15^,^16^,^17^, on the previously described synergistic effects by L-ala,*S*_P_ prodrugs in hiPSC-CM syncytia on FP rate and IMP amplitude measured in the CardioECR platform. To maximize the effects of CatA inhibitors, hiPSC-CM syncytia were pre-incubated with these reagents for 30 minutes prior to a second addition at time = 0, alone or combined with other test agents. Figure 5a shows the results of this treatment with 3 μM or 10 μM Ebel, on the normalized, time-dependent effects on FP rate (top) and IMP amplitude (bottom) in spontaneously beating hiPSC-CM syncytia by 3 μM sofosbuvir, alone or co-administered with 0.3 μM amiodarone. Figure 5b shows the results of this treatment with 3 μM Ebel, on the normalized, time-dependent effects on FP rate and IMP amplitude in spontaneously beating hiPSC-CM syncytia by 0.3 μM MNI-1, alone or co-administered with 0.3 μM amiodarone. Figure 5c shows the results of this treatment with 30 μM SAR164653, on the normalized time-dependent effects on FP rate and IMP amplitude in spontaneously beating hiPSC-CM syncytia by 1 μM MNI-1, alone or co-administered with 0.3 μM amiodarone. Overall, Ebel demonstrated a concentration-dependent exacerbation of the effects associated with L-ala,*S*_P_ prodrug + amiodarone. SAR164653 showed a similar effect on L-ala,*S*_P_ prodrug MNI-1, suggesting this exacerbation can be attributed to a marked CatA inhibitory activity. Monitoring baseline IMP during all these studies revealed no evidence of generalized cytotoxity.

We conducted complementary measurements of intracellular concentrations of sofosbuvir prodrug, cleavage intermediate metabolite, and NTP metabolite in hiPSC-CMs. Ebel transiently inhibited the formation of the sofosbuvir cleavage intermediate metabolite to levels below the limit of quantitation after 30 min incubation (Supplementary Table 3). Similarly, CatA inhibitor SAR164653 decreased, with concentration and time dependence, the concentration of sofosbuvir cleaveage intermediate metabolite intermediate (Supplementary Table 4). Therefore, while CatA inhibitors reduced, as expected, L-ala,*S*_P_ prodrug cleavage intermediate metabolite levels, they did not reduce, but rather, exacerbated the observed FP rate and IMP amplitude effects in spontaneously beating hiPSC-CMs.

### Comparison of MNI-1 and MNI-4 effects on Ca^2+^ influx in HEK-293 cells heterologously expressing Ca_v_1.2 or Ca_v_1.3 channels

**Figure 6:**
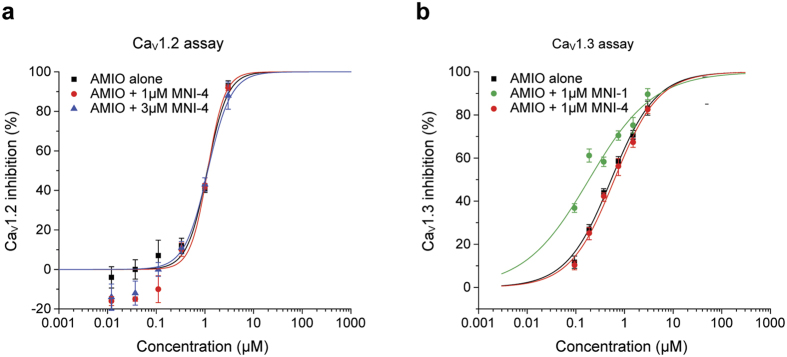
Lack of effect of MNI-4 on Ca^2+^ influx inhibition produced by amiodarone. (**a**) Lack of effect of MNI-4 on the Ca^2+^ influx inhibition produced by amiodarone on Ca_v1.2_/HEK-293 cells. (**b**) Effect of MNI-1 and lack of effect of MNI-4 on the Ca^2+^ influx inhibition produced by amiodarone on Ca_v1.3_/HEK-293 cells. IC_50_ values = 0.53 μM, 0.62 μM, and 0.19 μM for amiodarone alone, 1 μM MNI-4 + amiodarone, and 1 μM MNI-1 + amiodarone, respectively.

In a recently published study, we established a link to an intracellular Ca^2+^-handling mechanism in hiPSC-CM syncytia, and have shown that Ca^2+^ influx in Ca_v_1.2 /HEK-293 cells similarly models this synergistic effect^5^. We reported shifts in the concentration response of Ca^2+^ influx inhibition by amiodarone alone with L-ala,*S*_P_ pronucleotide MNI-1 co-applied at 1 μM and 3 μM (IC_50_ values shifting from 0.79 μM, to 0.47 μM and 0.29 μM, respectively). In the current study, we extend the findings of diastereoisomeric specificity into the Ca_v_1.2/HEK-293 cell line model, by examining the effects of the diastereoisomeric counterpart of MNI-1 (L-ala,*S*_P_), designated as MNI-4 (D-ala,*R*_P_), on the concentration-dependent, Ca^2+^ influx-inhibition by amiodarone. The IC_50_ value of MNI-1 alone in this model is >150 μM (21% inhibition at maximal tested concentration = 150 μM), while that of MNI-4 is approximately 36 μM (27–45 μM in repeat tests, n = 3 for each test). Amiodarone, in contrast, potently inhibits Ca_v_1.2 Ca^2+^ influx with IC_50_ value approx. 1 μM. Figure 6a illustrates the inability of 1 μM or 3 μM MNI-4 to produce a leftward shift on the inhibitory activity of amiodarone (IC_50_ = 1.1 μM for all 3 conditions). In addition, we directly compared MNI-1 and MNI-4 on the inibitory activity of amiodarone on Ca_v_1.3/HEK-293 influx. Figure 6b, illustrates diastereoisomeric specificity on the inhibitory activity of amiodarone on Ca_v_1.3 Ca^2+^ influx.

## Discussion

The major finding in this study was the lack of DDI with amiodarone shown by D-ala,*R*_P_ diastereoisomeric phosphoramidate HCV-NS5B prodrugs, including MK-3682, in preclinical *in vitro* and *in vivo* models. This finding was associated with the lack of metabolic activation for all D-ala,*R*_P_ prodrugs tested in hiPSC-CMs, and for MNI-2, in comparison to sofosbuvir, in guinea-pig hearts. Three pairs of diastereoisomeric prodrugs, including sofosbuvir (L-ala,*S*_P_) vs. MNI-2 (D-ala,*R*_P_), MNI-1 (L-ala,*S*_P_) vs. MNI-4 (D-ala,*S*_P_), and MNI-3 (L-ala,*S*_P_) vs. our company’s development compound MK-3682 (D-ala,*R*_P_), were compared in hiPSC-CMs. We have previously reported the paradoxical increase in beating rate produced by sofosbuvir and MNI-1, and the concomitant decrease in beat amplitude in this *in vitro* model^5^. In these studies, we have documented the reproducible, diastereoisomer-specific effect of phosphoramidate HCV-NS5B prodrugs with varying substituents in the 2′ position of the ribose moiety. In addition, we compared the metabolic activation of these compounds, noting that none of the D-ala,*R*_P_ prodrugs produced measurable cleavage intermediate or NTP metabolites in the cardiac tissues and hiPSC-CMs examined. We also noted that, in terms of the specific DDI with amiodarone, the intracellular measurements did not reveal large differences in the concentration of cleavage metabolite or NTP formed in the absence or presence of amiodarone, proportional with the observed pharmacological effects. We showed that a specific configuration at both diastereoisomeric centers of the phosphoramidate prodrugs, at the alanyl- and phosphoryl- groups (L-ala,*S*_P_), is required for the effects reported in spontaneously beating hiPSC-CM syncytia, and found reduced, yet measurable cleavage intermediate metabolite accumulation for the L-ala,*R*_P_ prodrug, despite its lack of effects in spontaneous beating activity.

The present studies suggest that cleavage metabolites produced by CatA or other CES enzymes active in cardiomyocytes do not play a major role in the adverse DDI with amiodarone. Although there is redundancy in the carboxylesterases that may play a role in the activation of phosphoramidate prodrugs in cardiomyocytes^8^,^18^, expression data and additional evidence argue for a relevant action by CatA^19^. While CatA inhibitors (Ebel and SAR164653) showed expected inhibitory effects on the formation of cleavage intermediate metabolite in hiPSC-CMs, they did not attenuate the effects on FP rate and IMP amplitude, as could have been expected, but rather exacerbated these effects, consistent with increased prodrug levels with CatA inhibition. Together, the observations reported in this study emphasize the specific role of L-ala,*S*_P_ prodrug in the adverse effects associated with the amiodarone DDI. Additional information, some of it previously reported^5^, include: fast onset of effect associated with the DDI on hiPSC-CM Ca^2+^ transients (FDSS) and FP rate (CardioECR); fast washout of FP rate effect (data not shown); fast effects associated with the DDI on Ca^2+^ influx and Ca^2+^ currents in heterologous expression systems. An alternative mechanism for the effect could be an uncharacterized metabolite produced in a stereospecific manner.

We have recently reported the hemodynamic effects of sofosbuvir and MNI-1 in the presence of amiodarone, in anesthetized guinea pigs and in conscious rhesus monkeys, and the lack of these adverse effects by MK-3682^7^. With the current studies, we extend the lack of *in vivo* effects previously reported for MK-3682 to MNI-2, the D-ala,*R*_P_ counterpart of MNI-1. IV administration of MNI-2 alone or with amiodarone did not result in greater than additive decreases in HR in anesthetized guinea pigs or conscious rhesus monkeys. MNI-2 parent prodrug and MNI-2 cleavage intermediate metabolite plasma concentrations were similar in anesthetized guinea pigs treated with MNI-2 and MNI-2+amiodarone. Likewise, MNI-2 parent prodrug cardiac tissue concentrations were similar in guinea pigs treated with either MNI-2 or MNI-2 + amiodarone. However, both the MNI-2 cleavage intermediate metabolite and MNI-2 NTP metabolite were below the limit of quantification in guinea-pig hearts treated with either MNI-2 alone or the combination of MNI-2 and amiodarone. In contrast, cleavage intermediate metabolite and NTP were present in hearts from sofosbuvir and sofosbuvir + amiodarone treated guinea pigs (Supplementary Fig. 1). Thus, the preclinical *in vivo* findings support the systematic findings in the hiPSC-CM syncytia model, while providing correct directionality (bradycardia vs increased FP rate *in vitro*), as a translational anchor predictive of clinical cardiac safety for MK-3682 and other D-ala,*R*_P_ phosphoramidate prodrugs.

The stereospecificity associated with adverse cardiac effects in our preclinical *in vitro* and *in vivo* models was also shown to extend to the simpler, Ca^2+^ channel overexpression system; a model we previously demonstrated to be sensitive to L-ala,*S*_P_ + amiodarone-dependent effects exemplified by MNI-1^5^. In this study, we showed that MNI-4, the D-ala,*R*_P_ counterpart of MNI-1, fails to produce a leftward shift in the inhibitory effect of amiodarone, in contrast to MNI-1. This diastereoisomeric specificity was not only revealed on influx across Ca_v_1.2, the predominant L-type Ca^2+^ channel in cardiomyocytes, but also on influx across the Ca_v_1.3 isoform, specific to SA nodal cells^20^,^21^,^22^. The important role of Ca_v_1.3 channels on sinus rhythm has been established, not only on mouse knockout models phenotypically exhibiting bradycardia^20^, but also in human Ca_v_1.3 channelopathies associated with bradycardia and congenital deafness^22^,^23^. Together, these findings confirm our previously reported results in Ca_v_1.2 HEK293 overexpressing cells, and provide additional insights into an ion flux-based mechanism behind the severe bradycardia associated with these L-ala,*S*_P_ HCV-NS5B DAAs and amiodarone. In hiPSC-CMs, we hypothesize a role for intracellular Ca^2+^ in the DDI, evidenced by the action of agents affecting sarcoplasmic reticulum Ca^2+^ load (i.e.,ryanodine and thapsigargin) or NCX1 function (i.e. SEA 0400 and Ca^2+^ clearance) on the combined action of MNI-1 + amiodarone. In the simpler system of Ca_v_1.2 HEK293 cells, we dissected the DDI on Ca_v_1.2 channels, as a shift by MNI-1 in the inhibitory effect of amiodarone on calcium influx in intact cells, and further characterized it as a shift in the inhibition by amiodarone of Ca_v_1.2 channel inactivation in whole cell patch clamp experiments using the perforated patch technique. We hypothesize a role for intracellular Ca^2+^ and L-type Ca^2+^ channel inactivation similar to that classically characterized in guinea pig ventricular myocytes^24^. Via Ca^2+^-sensitive inactivation, the L-type Ca^2+^ channel integrates both sarcoplasmic reticulum Ca^2+^ release, local and global Ca^2+^ entry, and membrane potential changes; the effect of the nucleoside in synergy with amiodarone may involve one of these mechanisms of the control of Ca^2+^ near the cytoplasmic face of the channel^25^. One could also envision a potential adverse effect of L-ala,SP prodrugs on hearing, through similar interactions with Cav1.3 channels in auditory hair cells. We explain the apparent inability by other investigators to detect DDI-associated effects on heterologously expressing Ca_v_1.2 cell lines^6^, primarily in terms of the voltage dependence of the effect, and potential methodological differences, as previously reported^5^.

Evidence of stereoselective metabolism by CatA and CES1 was detected for sofosbuvir and its L-ala,*R*_P_ counterpart in a replicon system^10^. In the current studies, we have revealed the importance of the L-ala,*S*_P_ vs. D-ala,*R*_P_ stereospecificity in determining adverse cardiac DDI effects. Our recently published data provided mechanistic insights and clinical translatability^5^,^7^, and also initial evidence that not all DAA HCV-NS5B agents exhibit this cardiac DDI^7^. This study shows that the D-ala,*R*_P_ configuration, exemplified by MK-3682, MNI-2, and MNI-4, does not present the adverse pharmacodynamic interactions detected with the L-ala,*S*_P_ pronucleotide diastereochemistry, exemplified by sofosbuvir, MNI-1, and MNI-3, in cardiomyocytes. In addition, the D-ala,*R*_P_ pronucleotides appear metabolically inactive toward NTP conversion in cardiomyocytes. MK-3682 has been shown to be active in patients infected with the human hepatitis C virus and treated with MK-3682 alone or in combination with other HCV viral therapies^26^,^27^.

## Methods

### Drugs

Diastereoisomeric pairs of HCV-NS5B inhibitor prodrugs were synthesized in-house for research purposes, as follows: phosphoramidate L-ala,*S*_P_ or D-ala,*R*_P_ 2′-fluoro-2′-methyl ribose “substituted nucleotide prodrug (i.e., sofosbuvir: isopropyl (2S)-2-[[[(2R,3R,4R,5R)-5-(2,4-dioxopyrimidin-1-yl)-4-fluoro-3-hydroxy-4-methyl-tetrahydrofuran-2-yl]methoxy-phenoxy-phosphoryl]amino]propanoate, or MNI-2: isopropyl (2R)-2-[[[(2R,3R,4R,5R)-5-(2,4-dioxopyrimidin-1-yl)-4-fluoro-3-hydroxy-4-methyl-tetrahydrofuran-2-yl]methoxy-phenoxy-phosphoryl]amino]propanoate); phosphoramidate L-ala,*S*_P_ or D-ala,*R*_P_ 2′-alkyne-2′-methyl ribose substituted nucleotide prodrug (i.e., MNI-1: isopropyl (2S)-2-[[[(2R,3R,4R,5R)-5-(2,4-dioxopyrimidin-1-yl)-4-ethynyl-3-hydroxy-4-methyl-tetrahydrofuran-2-yl]methoxy-phenoxy-phosphoryl]amino]propanoate, or MNI-4: isopropyl (2R)-2-[[[(2R,3R,4R,5R)-5-(2,4-dioxopyrimidin-1-yl)-4-ethynyl-3-hydroxy-4-methyl-tetrahydrofuran-2-yl]methoxy-phenoxy-phosphoryl]amino]propanoate); phosphoramidate L-ala,*S*_P_ or D-ala,*R*_P_ 2′-chloro-2′-methyl ribose substituted nucleotide prodrug (i.e., MNI-3: isopropyl (2S)-2-[[[(2R,3R,4R,5R)-4-chloro-5-(2,4-dioxopyrimidin-1-yl)-3-hydroxy-4-methyl-tetrahydrofuran-2-yl]methoxy-phenoxy-phosphoryl]amino]propanoate, or MK-3682: isopropyl (2R)-2-[[[(2R,3R,4R,5R)-4-chloro-5-(2,4-dioxopyrimidin-1-yl)-3-hydroxy-4-methyl-tetrahydrofuran-2-yl]methoxy-phenoxy-phosphoryl]amino]propanoate). Table 1 shows the designation and chemical structure of all the phosphoramidate prodrugs compared as diastereochemical pairs in this study. In addition, “mixed” stereoisomers L-ala,*R*_P_ or D-ala,*S*_P_ 2′-fluoro-2′-methyl ribose substituted nucleotide prodrugs were synthesized in-house for additional mechanistic studies, and designated as MNI-5 or MNI-6, respectively (Supplementary Table 1).

Amiodarone used for *in vitro* studies was purchased from Sigma-Aldrich (St. Louis, MO, USA). Amiodarone used for *in vivo* studies was obtained as the clinical IV formulation from Mylan Laboratories (NDC 67457-153-18) and diluted with 5% dextrose as needed. Ebelactone B was purchased from Enzo Life Sciences, Inc. (Farmingdale, NY, USA) and Santa Cruz Biotechnology, Inc. (Dallas, TX, USA). CatA inhibitor SAR164653 (also known as compound 2a, or SAR1)^15^,^16^,^17^ was synthesized in house for research purposes.

### RTCA Cardio and RTCA CardioECR studies

hiPSC-CMs (iCells^®^) from Cellular Dynamics International (CDI, Madison, WI, USA) were seeded onto 48-well CardioECR or 96-well Cardio E-Plates^®^ (ACEA Biosciences Inc., San Diego, CA, USA) coated with 10 μg/mL fibronectin (Sigma Aldrich, Catalog# F1141) at 30,000 cells/well, following manufacturer’s recommendations. Cells were maintained in culture (37 °C, 5% CO_2_) for a period of 14 days with iCell Maintenance^®^ media (CDI, Madison, WI, USA) exchanged every 2–3 days. Compound addition was only performed on or after Day 14 following cell seeding.

Compound stock solutions were prepared in 100% DMSO or H_2_O. On the day of compound addition, the media was exchanged with fresh iCell Maintenance^®^ media and allowed to equilibrate for at least 3 h in the incubator. The plates were read on an xCELLigence^®^ RTCA CardioECR or RTCA Cardio (ACEA Biosciences Inc., San Diego, CA, USA). Control pre-reads to establish a baseline were recorded for at least 45 minutes (4 reads at 15-min intervals) prior to compound addition. The compound stock solutions were diluted into iCell Maintenance^®^ media and quickly added to the plate. The plate was continuously monitored for at least 18 h following compound addition. IMP data were sampled at 12 ms (83 Hz), while FP rate data were collected at 0.1 ms (10 KHz). Attachment, growth and viability of syncytia were monitored by means of the baseline IMP signal, as previously described^28^. IMP and FP signals were only interpreted if the baseline IMP was maintained throughout the measurement period (usually ≤18 hrs) at ≥70% of the value before test compound application (pre-read value).

### HEK-293 /Ca_v1.2_ or Ca_v1.3_ assay

The HEK-293 cell line overexpressing Ca_v_1.2 channel proteins was maintained in-house. HEK-293 cells transiently overexpressing Ca_v_1.3 channel proteins were purchased from ChanTest (Charles River Laboratories, Cleveland, OH, USA). The assay was conducted as previously described^5^. Briefly, on experiment day, cells were incubated with Codex ACTOne^®^ dye (Codex Biosolutions, Inc., Gaithersburg, MD, USA) formulated in PPB buffer containing 25 mM potassium (in mM: 127 NaCl, 25 KCl 0.005 CaCl_2_, 1.7 MgCl_2_, 10 HEPES, pH = 7.2 with NaOH), or PPB buffer containing 1 mM potassium (in mM: 151 NaCl, 1 KCl 0.005 CaCl_2_, 1.7 MgCl_2_, 10 HEPES, pH = 7.2 with NaOH) for 1 h at room temperature, then test compounds were added for another 30-minute incubation at room temperature with a final volume of 100 μL. The Hamamatsu FDSS/μCell imaging platform simultaneously collected Ca^2+^ signals from 96-well plates, at a sampling rate of 16 Hz for 20 seconds as baseline, then a trigger buffer (containing in mM: 119 NaCl, 25 KCl, 4 CaCl_2_, 1.7 MgCl_2_, 10 HEPES, pH = 7.2 with NaOH) was added using the dispenser of the FDSS/μCell instrument to generate Ca^2+^ transient for 40 seconds. The peak amplitude within the latter 40 seconds minus the average amplitude of the first 20 seconds is the final Ca^2+^ transient response of each well. Average responses from wells treated with 10 μM nifedipine (reference CCB) was used as 100% inhibition (R_max_); and average responses from wells treated with 0.1% DMSO was set as 0% inhibition (R_min_). Relative response of each well was calculated as follows:


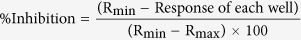


### Measurement of intracellular prodrug, cleavage intermediate metabolite, and NTP metabolite concentrations

Approximately 18 h before dosing, iCell hiPSC-CMs were switched to iCell cardiomyocyte maintenance media free of Penicillin/Streptomycin. Cells were exposed to vehicle control (0.1% DMSO) or 10 μM of each HCV-NS5B prodrug prepared in the same maintenance media for 0.5 h, 1.5 h, 4 h, and 18 h. A separate set of cells were dosed with 10 μM of prodrug + 0.3 μM amiodarone (final DMSO concentration in all dosing conditions were 0.1%). After the corresponding treatment, cells were washed 2x with 200 μL of ice-cold magnesium- and calcium-free PBS buffer. Cells were then lysed in 100 μL of ice-cold lysis solution (70/30 methanol/water containing 20 mM EDTA and 20 mM EGTA, pH 8) by pipetting up and down 5 times. Plates were sealed and transferred on dry ice for determination of prodrug, cleavage intermediate metabolite, and NTP metabolite concentrations. Intracellular prodrug concentrations were measured using a reversed phase HPLC- high resolution mass spectrometry method. A dimethylhexylamine based ion-pairing HPLC-MS/MS method was used to measure intracellular cleavage intermediate metabolite and NTP metabolite concentrations. Intracellular concentrations (pmol/million cells) of prodrug, cleavage intermediate metabolite, and NTP metabolite were quantitated against calibration standards of the same analytes prepared from control iCell hiPSC-CM lysates. The structures of each of these species are presented in Table 1.

For prodrug analyses, full scan high resolution mass spectrometry data was collected using an Exactive™ Orbitrap mass spectrometer with an electrospray source operated in the positive ion mode. Prodrug chromatograms were extracted from full scan high resolution mass spectrometry data for the masses indicated in Supplementary Table 5. Absence of endogenous interference(s) was verified by extracting the same ion from full scan high resolution mass spectrometry chromatograms of unspiked control cell lysates. For the cleavage intermediate metabolite and NTP metabolite an electrospray MS/MS method run on a SCIEX API 4000 mass spectrometer with turboV ionspray source was used in negative ion mode monitoring the transitions indicated in Supplementary Table 5. Absence of endogenous interference(s) was verified by monitoring of the same MRM transitions for unspiked control cell lysates. Measured intracellular prodrug, cleavage intermediate metabolite, and NTP metabolite concentrations (in pmol/million cells units) were tabulated (Table 2).

### *In vivo* Studies

All animal studies were conducted in accord with the Guide for the Care and Use of Laboratory Animals (Institute of Laboratory Animal Resources, Commission on Life Sciences, National Research Council, 2011) and were approved by the Institutional Animal Care and Use Committee at MRL, Merck & Co. (West Point, PA).

### Anesthetized guinea pigs

Adult, male Dunkin-Hartley guinea pigs (350–550 g) were anesthetized and instrumented as previously described^7^,^29^. The effects of HCV prodrug alone or in combination with amiodarone on arterial BP, HR, electrocardiogram (ECG) intervals were then evaluated in separate studies, as follows: MNI-2 (n = 4) or amiodarone (n = 5) was administered separately as 4 sequential 30-min IV infusions of 4, 2, 2 and 2 mg/kg (cumulative dose of 10 mg/kg). Then, these doses of MNI-2 were co-administered with IV amiodarone (1, 0.5, 0.5, 0.5 mg/kg/30 min (n = 6). The effects of the vehicle alone (30% Captisol^®^, 1 mL/kg every 30 minutes, n = 6) and amiodarone (n = 5) were previously reported^7^, and summary data from that study were plotted to provide comparisons.

### Conscious, restrained rhesus monkeys

Arterial BP and ECG waveforms from four chronically-instrumented male, rhesus monkeys (8-16 kg at time of study, approximately 5–8 years of age), were continuously recorded (Notocord-hem™ v 4.3.0.67) and analyzed as described^7^. Prior to study, animals were acclimated to the restraint chair and the laboratory environment over multiple sessions. At the time of study, animals were weighed, brought to the laboratory in restraint chairs, and following equilibration administered either MNI-2 alone (4 mg/kg/30 min, then 2 mg/kg/30 min), or an IV pretreatment with amiodarone (3 mg/kg/30 min) up to 30-min prior administration of the same dose of MNI-2. MNI-2 was formulated in 30% Captisol^®^. Since the animals used for these studies were also used previously for studies on HCV-NS5B prodrugs^7^ and the studies were completed in a similar time frame, vehicle and amiodarone data presented were adapted from data published by Regan et al^7^. Blood samples (~0.75 mL per time point) were collected in EDTA tubes at the end of each infusion of MNI-2 and an additional sample was collected at the end of the baseline recording for animals pre-administered amiodarone. Samples were placed on wet ice, centrifuged at 4 °C and plasma removed and stored at −70 °C until analysis of concentration of test agents and any metabolites of interest.

## Additional Information

**How to cite this article:** Lagrutta, A. *et al*. Cardiac drug-drug interaction between HCV-NS5B pronucleotide inhibitors and amiodarone is determined by their specific diastereochemistry. *Sci. Rep.* **7**, 44820; doi: 10.1038/srep44820 (2017).

**Publisher's note:** Springer Nature remains neutral with regard to jurisdictional claims in published maps and institutional affiliations.

## Supplementary Material

Supplementary Data

## Figures and Tables

**Table 1:**
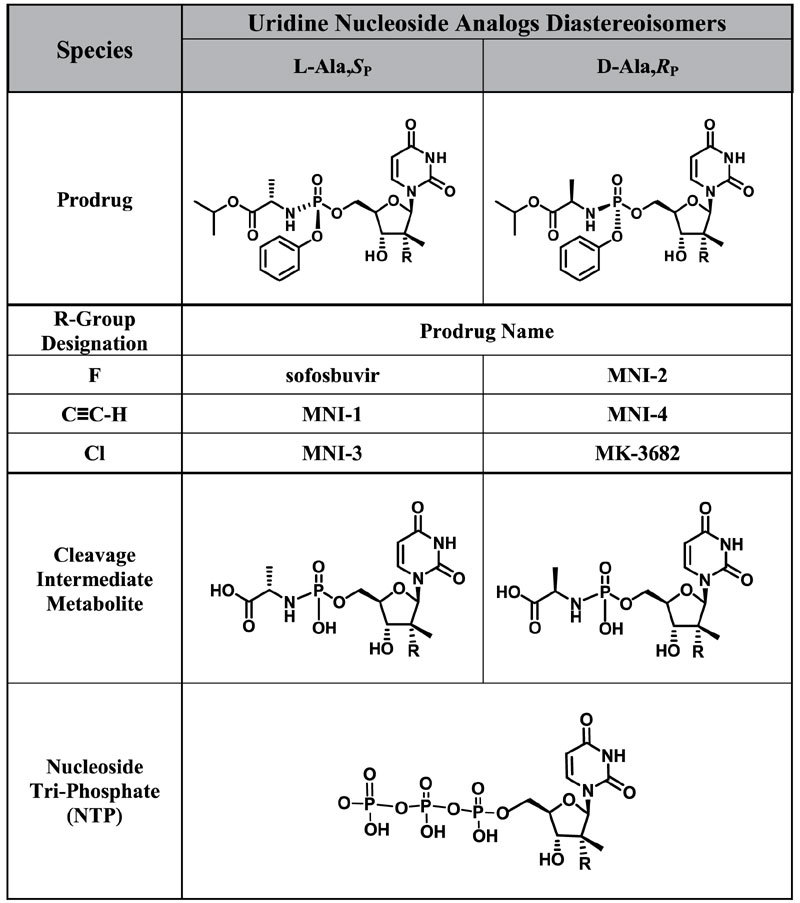
Prodrugs, cleavage intermediate metabolites, and NTP metabolites studied.

**Table 2:** Summary of prodrugs, cleavage intermediate metabolites, and NTP metabolites in. hiPSC-CMs after 30 min and 4 hours incubations.

Phosphoramidate Diastereochemistry	Species	Intracellular Concentration (pmol/million cells)			
		30 Min		4 Hours	
		Prodrug	Prodrug + Amiodarone	Prodrug	Prodrug + Amiodarone
L-ala,*S*P	**Sofosbuvir**				
	Prodrug	46.7 ± 3.7	45.6 ± 2.0	50.1 ± 2.1	58.4 ± 6.0
	Cleavage Intermediate Metabolite	57.3 ± 3.1	53.1 ± 2.2	564.7 ± 26.3	501.3 ± 24.2
	NTP Metabolite	BLOQ*	BLOQ	31.1 ± 2.2	24.8 ± 1.8
	**MNI-3**				
	Prodrug	97.3 ± 14.0	89.8 ± 12.5	106.9 ± 10.1	94.0 ± 6.0
	Cleavage Intermediate Metabolite	714.3 ± 34.7	596.7 ± 15.0	5796.7 ± 609.3	4560.0 ± 416.1
	NTP Metabolite	BLOQ	BLOQ	91.6 ± 6.2	65.1 ± 7.5
	**MNI-1**				
	Prodrug	75.4 ± 2.3	69.9 ± 5.3	69.1 ± 6.8	70.5 ± 14.4
	Cleavage Intermediate Metabolite	93.2 ± 2.1	91.1 ± 4.7	914.0 ± 12.3	719.7 ± 14.5
	NTP Metabolite	BLOQ	BLOQ	Detected but BLOQ	Detected but BLOQ
	**MNI-2**				
	Prodrug	53.5 ± 4.5	54.8 ± 3.3	173.0 ± 133.3	61.0 ± 3.3
D-ala,*R*P	Cleavage Intermediate Metabolite	BLOQ	BLOQ	BLOQ	BLOQ
	NTP Metabolite	BLOQ	BLOQ	BLOQ	BLOQ
	**MK-3682**				
	Prodrug	89.8 ± 12.9	95.0 ± 6.7	100.4 ± 13.2	91.9 ± 12.4
	Cleavage Intermediate Metabolite	BLOQ	BLOQ	BLOQ	BLOQ
	NTP Metabolite	BLOQ	BLOQ	BLOQ	BLOQ
	**MNI-4**				
	Parent Prodrug	75.4 ± 5.1	84.8 ± 12.4	77.4 ± 6.7	78.7 ± 6.4
	Cleavage Intermediate Metabolite	BLOQ	BLOQ	BLOQ	BLOQ
	NTP Metabolite	BLOQ	BLOQ	BLOQ	BLOQ

*BLOQ – Below the limit of quantification (17 pmol/million cells).
