# Supplementary material for: Cardiac drug-drug interaction between HCV-NS5B pronucleotide inhibitors and amiodarone is determined by their specific diastereochemistry
Source: Sci Rep. 2017 Mar 22;7:44820. doi: 10.1038/srep44820 (PMC5361079; doi:10.1038/srep44820)
Supplement: Supplementary Data [file srep44820-s1.pdf]

Cardiac drug-drug interaction between HCV-NS5B pronucleotide inhibitors and amiodarone is determined by their specific diastereochemistry

Armando Lagrutta[1,]* Christopher P. Regan[1], Haoyu Zeng[1], John P. Imredy[1], Kenneth Koeplinger[2], Pierre Morissette[1], Liping Liu[3], Gordon Wollenberg[4], Christopher Brynczka[5], José Lebrón[3], Joseph DeGeorge[6], Frederick Sannajust[1]

SUPPLEMENTARY MATERIALS

[1] Dept. Safety and Exploratory Pharmacology, Safety Assessment and Laboratory Animal Resources, Merck Research Laboratories, West Point, PA, USA.
[2] Dept. Preclinical ADME, Pharmacokinetics, Pharmacodynamics and Drug Metabolism, Merck Research Laboratories, West Point, PA, USA.
[3] Dept. Investigative Laboratory Sciences, Safety Assessment and Laboratory Animal Resources, Merck Research Laboratories, West Point, PA, USA.
[4] Dept. Pathology, Safety Assessment and Laboratory Animal Resources, Merck Research Laboratories, West Point, PA, USA.
[5] Dept. Program Development, Safety Assessment and Laboratory Animal Resources, Merck Research Laboratories, West Point, PA, USA.
[6] Safety Assessment and Laboratory Animal Resources, Merck Research Laboratories, West Point, PA, USA.
* Correspondence and requests for materials should be addressed to A.L. (armando_lagrutta@merck.com).

Supplementary Table 1 **Intracellular concentrations of prodrug, cleavage intermediate metabolite, and NTP metabolite at 30 min and 4 hours post incubations of 10 µM mixed stereoisomer L-ala,Rp or D-ala,Sp in hiPSC-CMs**

Species	Intracellular Concentration (pmol/million cells)			
	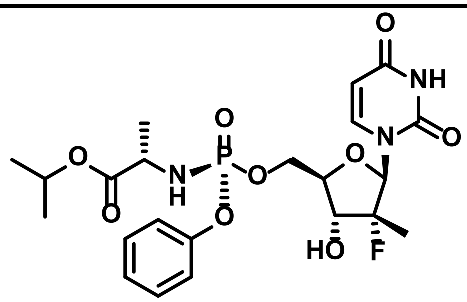		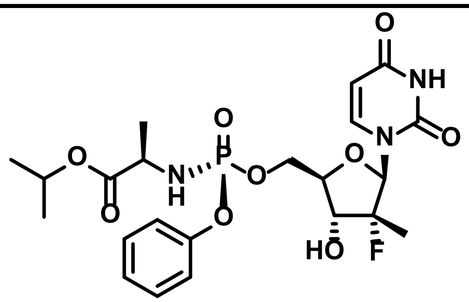	
	L-ala,Rp (MNI-5)		D-ala,Sp (MNI-6)	
	30 Min	4 Hours	30 Min	4 Hours
**Prodrug**	**53.6 ± 14.2**	**97.1 ± 36.8**	**46.6 ± 3.1**	**65.5 ± 5.8**
**Cleavage Intermediate Metabolite**	**BLOQ**	**121.0 ± 6.24**	**BLOQ**	**BLOQ**
**NTP Metabolite**	**BLOQ**	**BLOQ**	**BLOQ**	**BLOQ**

BLOQ= below limit of quantitation: LOQ=17 pmol/million cells

Supplementary Table 2 Mean ± SD plasma concentration (µM) of MNI-2, MNI-2 primary cleavage metabolite, and AMIO in anesthetized guinea pigs

Plasma concentration (Mean ± SE in µM)	End of Infusion 1	End of Infusion 2	End of Infusion 3	End of Infusion 4
**Treatment: AMIO + vehicle (n=5)**				
AMIO	1.1 ± 0.2	1.1 ± 0.3	1.1 ± 0.3	1.4 ± 0.4
**Treatment: MNI-2 + vehicle (n=4)**				
MNI-2	4.2 ± 2.1	4.2 ± 0.9	3.9 ± 0.1	3.7 ± 1.0
MNI-2 cleavage metabolite	0.2 ± 0.2	0.2 ± 0.04	0.4 ± 0.08	0.4 ± 0.4
**Treatment: MNI-2+AMIO (n=6)**				
AMIO	1.2 ± 0.2	0.9 ± 0.04	0.9 ± 0.1	0.9 ± 0.2
MNI-2	3.0 ± 0.8	3.5 ± 1.0	3.7 ± 0.6	3.7 ± 0.9
MNI-2 cleavage metabolite	0.4 ± 0.2	0.3 ± 0.1	0.6 ± 0.3	0.6 ± 0.3

Supplementary Table 3 Effect of CatA inhibitor ebelactone-B (Ebel) in the presence of 0.3 µM amiodarone on accumulation of sofosbuvir (SOF) prodrug, cleavage intermediate metabolite or NTP metabolite

Species	Intracellular Concentration (pmol/million cells)							
	30 Min				4 Hours			
	SOF-3 µM		SOF-10 µM		SOF-3 µM		SOF-10 µM	
	SOF	SOF + A + Ebel	SOF	SOF + A + Ebel	SOF	SOF + A + Ebel	SOF	SOF + A + Ebel
Sofosbuvir (Prodrug)	19.5 ± 0.9	32.3 ± 5.1	148.6 ± 119.9	53.4 ± 9.5	19.6 ± 5.8	46.9 ± 8.9	66.9 ± 6.8	71.5 ± 15.0
Cleavage Intermediate Metabolite	26.2 ± 2.5	BLOQ	59.1 ± 3.8	BLOQ	171.7 ± 2.5	187 ± 3.0	644.7 ± 23.1	334.7 ± 26.5
NTP Metabolite	BLOQ	BLOQ	BLOQ	BLOQ	BLOQ	BLOQ	37.8 ± 1.0	BLOQ

BLOQ= below limit of quantitation: LOQ=17 pmol/million cells

Supplementary Table 4 Effect of CatA inhibitor 2a (SAR164653) on accumulation of sofosbuvir (SOF) prodrug, cleavage intermediate metabolite or NTP metabolite

Species	Intracellular Concentration (pmol/million cells)									
	SOF-10 µM									
	30 Min					4 Hours				
	2a 0 µM	2a 1 µM	2a 10 µM	2a 50 µM	2a 100 µM	2a 0 µM	2a 1 µM	2a 10 µM	2a 50 µM	2a 100 µM
Sofosbuvir (Prodrug)	87 ± 19.4	41.5 ± 2.3	55.6 ± 2.7	70.2 ± 1.9	74.8 ± 3.3	78.4 ± 13.1	57.4 ± 4.2	73.3 ± 10.7	76.8 ± 11.7	92.8 ± 3.6
Cleavage Intermediate Metabolite	53.8 ± 7.5	48.1 ± 2.0	38.7 ± 4.6	34.1 ± 3.1	24.2 ± 5.1	536 ± 68.3	643.3 ± 34.9	429.3 ± 70.1	263.7 ± 52.0	218.3 ± 37.2
NTP Metabolite	BLOQ	BLOQ	BLOQ	BLOQ	BLOQ	37.3 ± 5.9	34.0 ± 4.8	25.8 ± 1.4	BLOQ	BLOQ

BLOQ= below limit of quantitation: LOQ=17 pmol/million cells

Supplementary Table 5 High resolution mass spectrometry masses for prodrugs, cleavage intermediate metabolites, and NTP metabolites in hiPSC-CMs after 30-min and 4-h incubations

Compound	Prodrug HRMS $[M+H]^+$	Cleavage Metabolite $[M-H]^-$ MS/MS	NTP Metabolite $[M-H]^-$ MS/MS
SOF	530.170 ± 15 ppm	410>150	499>159
MNI-2	530.170 ± 15 ppm	410>150	499>159
MNI-5	530.170 ± 15 ppm	410>150	499>159
MNI-6	530.170 ± 15 ppm	410>150	499>159
MNI-1	536.180 ± 15 ppm	416>150	505>159
MNI-4	536.180 ± 15 ppm	416>150	505>159
MNI-3	546.141 ± 15 ppm	426>150	515>159
MK-3682	546.141 ± 15 ppm	426>150	515>159

Supplementary Figure 1: Lack on concentration-dependent, steady-state effect by either L-ala,R_P prodrug (MNI-5) or D-ala,S_P prodrug (MNI-6), co-administered with 0.3 µM amiodarone, on parameters measured in spontaneously beating hiPSC-CM syncytia.

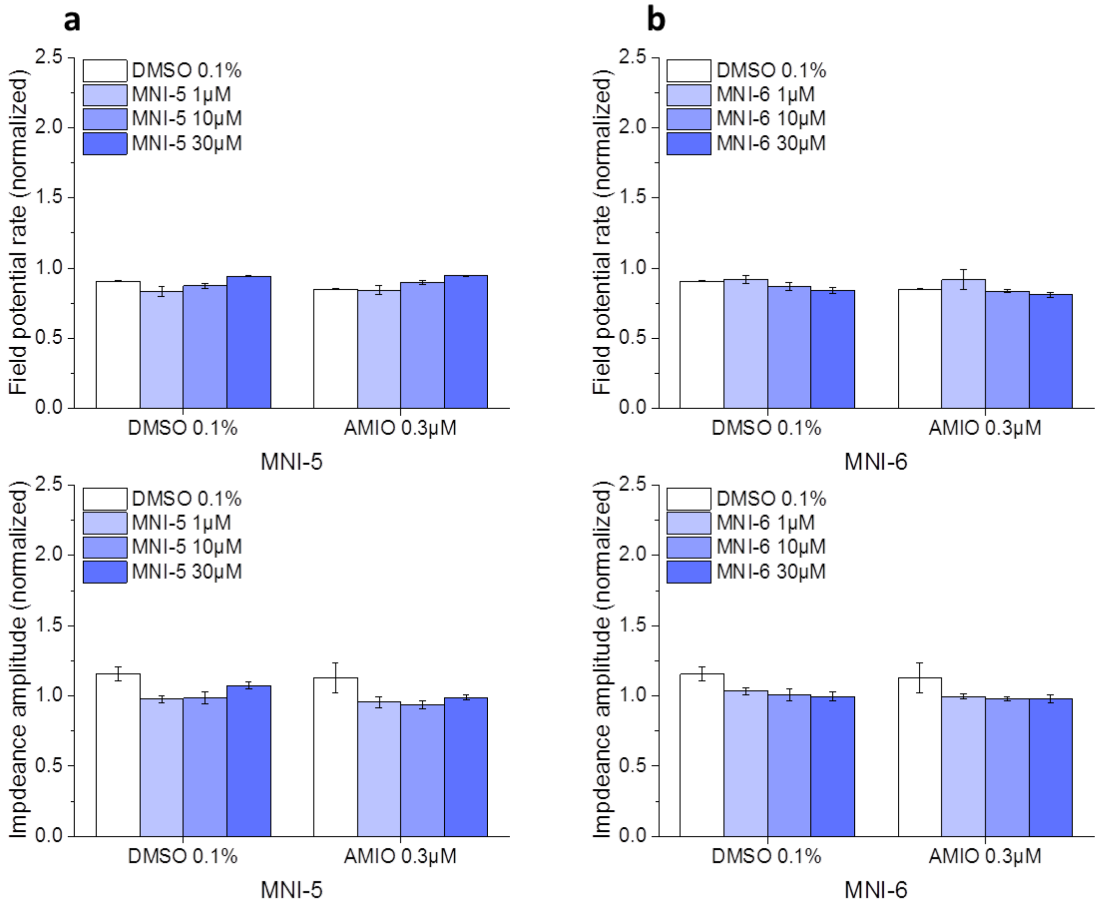

(a – b) Parameters measured: FP rate (top panels); IMP amplitude (bottom panels). Colored bar graphs in each panel show steady state effects produced by increasing concentrations of each prodrug ± amiodarone: (a) MNI-5 (L-ala,R_P). (b) MNI-6 (D-ala,S_P). Clear bar graphs illustrate normalized measurement with DMSO vehicle or amiodarone alone. Data are normalized to parameters measured at time = 0 (mean ± SEM, n=6).

Supplementary Figure 2: Concentrations of prodrugs, cleavage metabolites, and NTP in plasma and cardiac tissue extract from anesthetized guinea pigs after treatment.

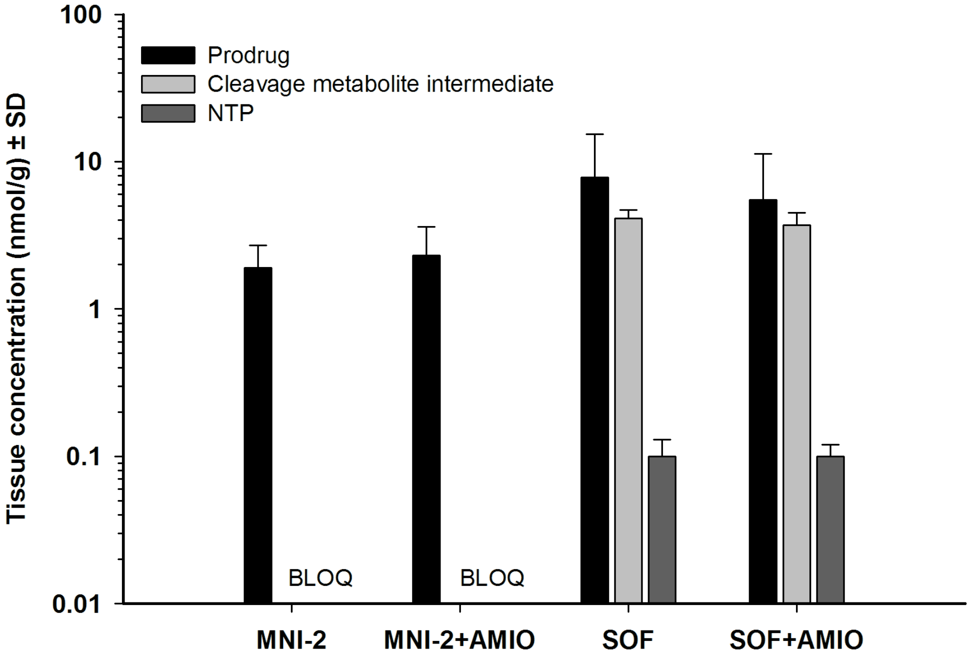

Tissue concentrations above the limit of quantitation were measured for MNI-2 (D-ala,R_P) and SOF prodrugs (L-ala,S_P), and to comparable levels in the presence or absence of amiodarone. Cleavage metabolite and NTP were below the limit of quantitation for MNI-2 (BLOQ). Tissues were collected immediately following a 2-h treatment, as described in Results.
